# How Does Meditation-Based Lifestyle Modification Affect Pain Intensity, Pain Self-Efficacy, and Quality of Life in Chronic Pain Patients? An Experimental Single-Case Study

**DOI:** 10.3390/jcm12113778

**Published:** 2023-05-31

**Authors:** Karin Matko, Meike Burzynski, Maximilian Pilhatsch, Benno Brinkhaus, Andreas Michalsen, Holger C. Bringmann

**Affiliations:** 1 Department of Psychology, Chemnitz University of Technology, 09120 Chemnitz, Germany; karin.matko@psychologie.tu-chemnitz.de; 2 Department of Psychiatry and Psychotherapy, Carl Gustav Carus University Hospital, Technische Universität Dresden, 01069 Dresden, Germany; 3 Department of Psychiatry and Psychotherapy, Elblandklinikum, 01445 Radebeul, Germany; 4 Institute of Social Medicine, Epidemiology, and Health Economics, Charité-Universitätsmedizin, Corporate Member of Freie Universität Berlin and Humboldt-Universität zu Berlin, 10117 Berlin, Germany; 5 Department of Internal and Integrative Medicine, Immanuel Hospital Berlin, 14109 Berlin, Germany; 6 Department of Psychiatry, Psychosomatics and Psychotherapy, Krankenhaus Spremberg, 03130 Spremberg, Germany

**Keywords:** chronic pain, yoga, meditation, ethics, pain intensity, self-efficacy, quality of life, individual differences, mind-body medicine, single-case research

## Abstract

Introduction: Chronic pain is a growing worldwide health problem and complementary and integrative therapy options are becoming increasingly important. Multi-component yoga interventions represent such an integrative therapy approach with a promising body of evidence. Methods: The present study employed an experimental single-case multiple-baseline design. It investigated the effects of an 8-week yoga-based mind-body intervention, Meditation-Based Lifestyle Modification (MBLM), in the treatment of chronic pain. The main outcomes were pain intensity (BPI-sf), quality of life (WHO-5), and pain self-efficacy (PSEQ). Results: Twenty-two patients with chronic pain (back pain, fibromyalgia, or migraines) participated in the study and 17 women completed the intervention. MBLM proved to be an effective intervention for a large proportion of the participants. The largest effects were found for pain self-efficacy (TAU-*U* = 0.35), followed by average pain intensity (TAU-*U* = 0.21), quality of life (TAU-*U* = 0.23), and most severe pain (TAU-*U* = 0.14). However, the participants varied in their responses to the treatment. Conclusion: The present results point to relevant clinical effects of MBLM for the multifactorial conditions of chronic pain. Future controlled clinical studies should investigate its usefulness and safety with larger samples. The ethical and philosophical aspects of yoga should be further explored to verify their therapeutic utility.

## 1. Introduction

Chronic pain affects between 35 and 50% of the global population [1,2,3] and represents the leading cause of years of life with disability and illness [4,5]. Chronic pain is not only a common, complex, and burdening problem for individuals but also poses significant socioeconomic challenges for society [3,6]. There is a great need for an integrative interdisciplinary management of chronic pain that addresses both patient-centered multimodal and societal levels [4,7].

Interdisciplinary multimodal pain therapy (IMPT) is considered the most important intervention for chronic pain relief. Its effectiveness has been confirmed in several studies [8,9], and it is based on a combination of medical interventions, psychotherapy, and exercise therapy. Its primary goal is not only to reduce pain but also to educate the affected person to gain a biopsychosocial understanding of the disease and restore their physical, psychological, and social functions despite the pain [10,11,12,13]. However, many programs are difficult to access, not integrative or patient-centered, and do not provide effective and, more importantly, long-term strategies for the overall treatment goals of IMPT. As a result, many patients are dissatisfied with conventional methods and, therefore, often turn to alternative treatments [14,15,16].

Yoga represents a successful mind-body medicine (MBM) practice that is safe, inexpensive, and effective in the complementary treatment of chronic pain [16,17,18]. Today, approximately 30 million people worldwide practice yoga regularly [19], and one of the most common reasons for practicing yoga is pain [20]. The growing amount of scientific research over the past decade has shown significant improvements in pain intensity, pain-related functional limitations, and wellbeing following yoga interventions [21,22,23,24]. In addition, yoga has been effective for people with chronic pain in alleviating concurrent depression, stress, and anxiety disorders and enhancing body awareness, pain acceptance, coping strategies, and self-efficacy [16,25,26,27]. The growing number of randomized controlled trials (RCTs) and reviews demonstrate the increasing therapeutic importance of yoga [19].

Nevertheless, there is a lack of high-quality studies, interventions are often poorly described, and the practice of yoga is often reduced to physical and breathing exercises without incorporating its ethical roots [19,28,29]. Blinding is often difficult, inducing the possibility of a placebo effect. Furthermore, inconsistent sample sizes and study durations and large methodological heterogeneity make it difficult to summarize study results in meta-analyses and translate them into meaningful outcomes [30,31]. In addition, Sharma et al. [31] criticized the limited informative value of RCTs and suggested that the inclusion of qualitative data may help to better understand the multidimensional effects of yoga on human mental and physical health.

To address some of these limitations, the present study used a single-case multiple-baseline design [32], which is very suitable for capturing individual effects and changes over time in greater detail. Furthermore, we employed the mind-body program Meditation-Based Lifestyle Modification (MBLM), which provides a consistent and comprehensive approach to classical yoga and incorporates its ethical component. It follows a prescribed structure (set duration for the yoga practice, meditation, and ethical teachings, which are taught in a psychotherapy-like setting) and engages participants to practice on their own [33]. The present study examined MBLM’s feasibility and effects on pain-related outcomes in patients suffering from various forms of chronic pain. The program has been shown to be feasible and effective in depressed patients as well as healthy individuals [34,35]. Therefore, we hypothesized that it would lead to improvements in pain intensity, pain self-efficacy, and quality of life in chronic pain patients.

## 2. Materials and Methods

### 2.1. Study Design

**Figure 1:**
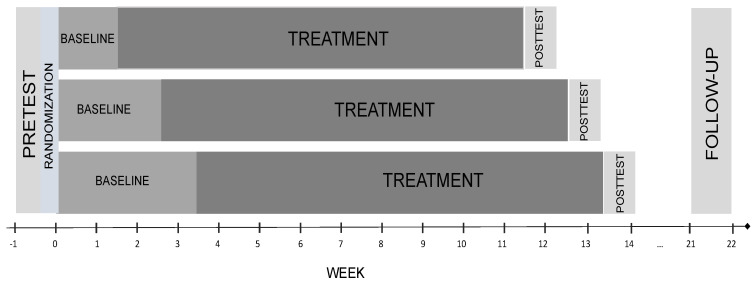
Study design.

All participants received the same treatment (MBLM), but they were randomly assigned to three baseline groups (10, 17, and 24 days). To keep the group sizes small, participants were split into two groups where treatments took place in the afternoon and evening of the same day. The treatment was delivered over eight weekly group sessions, but due to public holidays during the study, the total intervention length was 10 weeks. Each participant started the intervention according to their assigned baseline length. All measurements were taken online, and the pretest was completed one week before the beginning of the baseline phase. Baseline assessments started on the same day for all participants, and the participants received daily online questionnaires throughout the entire study period. Each participant received a posttest at the completion of the study and a follow-up 8 to 10 weeks later. Figure 1 depicts the study design.

### 2.2. Intervention

The MBLM program is a mind-body intervention based on the eightfold path of classical yoga that consists of 3 domains: ethical living, healthy lifestyle, and mantra meditation. It was delivered in 8 sequential 180 min group sessions that covered all three domains. The ethical living domain introduced participants to the philosophical aspects of classical yoga, presenting 1 of the 10 yamas and niyamas each week (the last 3 niyamas were covered in one session). The yamas and niyamas represent basic attitudes and practices in dealing with oneself and others, such as non-violence, truthfulness, contentment, self-discipline, etc. In the MBLM course, these are communicated to participants in a psychotherapeutic context in a culturally sensitive and application-oriented way. Furthermore, participants are invited to discuss these topics with the group and apply them to their everyday lives with the help of several ethical living activities (e.g., the practice of being truthful instead of “nice”). The healthy lifestyle domain included gentle physical exercises and breathing techniques, as well as general recommendations on diet and daily rhythms according to Ayurvedic medicine. Here, for example, the participants were advised to arise before 6 a.m. and follow a morning routine of personal hygiene, yoga, and meditation and eat their main meal at noon, use the last hours of the day for rest and relaxation, and go to bed no later than 10 p.m. In addition, general dietary recommendations were provided, highlighting the benefits of a plant-based diet with moderate use of natural sweeteners. During mantra meditation, the participants silently repeated a mantra for 20 to 25 min that they had chosen during an introductory session at the beginning of the treatment. The mantra served as an object of concentration to calm the mind and body. In deeper stages of meditation, the mantra could be abandoned and the concentration directed to inner silence. The participants were recommended to engage daily in 45 min of home practice covering the different domains. A detailed description of MBLM can be found in the work by Bringmann et al. [33].

### 2.3. Inclusion Criteria and Recruitment

The recruitment period was approximately three months between May and August 2019. To be included in the study, participants had to be outpatients who were at least 18 years old and had suffered from recurrent or persistent chronic pain for more than three months. They had to be in good enough physical condition to perform simple yoga exercises and sit still for approximately 20 min according to the self-assessment. Patients were excluded if they had obsessive-compulsive disorder, cerebrovascular disease, addictive disorders, psychotic symptoms, acute suicidality, severe multimorbidity, and yoga and meditation experience (>1 time per week in the past 6 months) or if they were currently participating in another yoga and meditation study. Participants were recruited via flyers and posters distributed in medical practices and chronic pain centers in Dresden and screened by MB through individual telephone calls. After screening 38 prospective participants, 26 of them were invited to attend one of two information events held by the authors. Twenty-two patients (twenty women and two men) decided to participate in the study and provided written consent. Participation was voluntarily and the participants received no financial compensation for their participation. During the study, five participants (including both men) dropped out for personal reasons (meditation difficulties, lack of interest, work overload, psychological problems, and depression). The final sample consisted of 17 female participants.

### 2.4. Measures

The instruments for daily and weekly measurements had to be suitable for a single-case experimental design, and thus, they had to be precise and short. All questionnaires were programmed and implemented using SoSci Survey [36] and were made available at www.soscisurvey.com. The data were collected between 2 September 2019 and 6 February 2020. All dependent variables were collected continuously throughout the study and during the pretest, posttest, and follow-up periods. Furthermore, the pretest assessed sociodemographic and clinical data on chronic pain, and the posttest and follow-up measured course satisfaction.

### 2.5. Pain

Daily pain was assessed using the German short form of the Brief Pain Inventory (BPI-sf) [37]. The BPI-sf is a 9-item questionnaire for the self-assessment of pain intensity and its impact on daily life. It contains questions about pain intensity over the past 24 h, current treatments, and their perceived effectiveness. In this study, we focus on the daily average and strongest pain participants reported in this questionnaire.

### 2.6. Pain Self-Efficacy

Pain self-efficacy was measured once each week using the German version of the Pain Self-Efficacy Questionnaire (PSEQ). The questionnaire consists of 10 items reflecting how confident a person feels that they can perform certain activities despite their pain. Items were rated on a 7-point Likert scale from 0 (not at all convinced) to 6 (completely convinced). The German version (FESS) is valid and has high internal consistency (α = 0.92) [38].

### 2.7. Wellbeing

Wellbeing was measured daily using the World Health Organization Wellbeing Index (WHO-5). The WHO-5 is a very short psychometric and generic self-assessment scale for measuring subjective well-being. It has high internal and external validity [39] and consists of five positively worded statements rated on a six-point Likert scale. High scores represent high subjective wellbeing. Due to the daily measurements, the time frame was adjusted from “in the last 2 weeks” to “in the last 24 h”.

### 2.8. Daily Experiences

The daily questionnaire included questions about the duration and subjective experiences with yoga, meditation, and ethical living. First, participants were asked to enter how many minutes they had practiced yoga and meditation on that day (0 if none) and whether they had engaged with the ethical living topic of the week (yes/no). Then, they were requested to indicate the experienced difficulty of each practice using a 5-point polarity profile ranging from 1 (very difficult) to 5 (very easy). In addition, we provided participants with a free text item in which they could describe any special events that they had experienced during that day.

### 2.9. Data Analysis

The data were analyzed visually and statistically by calculating the effect size TAU-*U*. To estimate the overall effect of the intervention, we performed a random-effects meta-analysis on each dependent variable. The results of these meta-analyses are presented visually using forest plots. If the results of all three analyses were consistent, then this would provide strong evidence for our findings. All statistical analyses were performed using R statistical software [40].

For the visual analysis, we generated individual dependent-variable-by-time plots using the package *lattice* [41]. Then, we assessed trends in the individual phases and level differences between the phases. The visual analysis followed the standards set by Kartochwill et al. [42] and Lane and Gast [43]. We focused on three components: trend, level, and stability. This allowed us to identify the individual effects of the intervention and provided a basis for the subsequent statistical analysis.

The TAU-*U* is a family of non-parametric estimates of effect size in single-case research designs. The effects of the intervention were calculated for each participant and for all three dependent variables using the package *scan* [44]. Then, the appropriate TAU-*U* coefficients were selected according to the recommendations of Parker et al. [45] and Brossart et al. [46]. Trends in the baseline phase (TAU-*U*_A vs. B−Trend A_), treatment phase (TAU-*U*_A vs. B+Trend B_), or both phases (TAU-*U*_A vs. B+Trend B−Trend A_) were corrected if they were visually prominent, larger than 0.40, or statistically significant (at *p* < 0.01). The sizes of the effects were determined following the guidelines set by Solomon et al. [47]. A TAU-*U* smaller 0.28 indicated a small effect, between 0.29 and 0.47 indicated a moderate effect, between 0.48 and 0.57 indicated a large effect, and 0.58 and larger indicated a very large effect.

The R package meta [48] was used to conduct a rudimentary meta-analysis for each dependent variable. Individual effect size estimates were plotted in forest plots with corresponding 95% confidence intervals (95% CI), and the overall effect was estimated using a random-effects model. If the 95% confidence intervals did not cross zero, the effect size estimates indicated a statistically significant positive or negative effect; if they crossed zero, they were non-significant [49]. Large differences in the locations and widths of the 95% confidence intervals indicated high heterogeneity and were measured by τ according to DerSimonian/Laird and *I*^2^ according to Higgins/Thompson. High heterogeneity was present when the value of *I*^2^ was greater than 75% [49].

## 3. Results

### 3.1. Study Population

The final study population included 17 female Caucasian outpatients of German nationality who were between 19 and 79 years old and mostly suffered from chronic back pain (with or without disc involvement), fibromyalgia, or chronic migraines. Some of the participants suffered from more than one pain-causing condition. In nearly half of the participants, the duration of the pain disorder was more than five years. An overview of the most important participant data can be found in Table 1.

### 3.2. Adherence

According to the daily responses, on average, the participants practiced yoga for 20.3 min (SD = 11.6 min) and mantra meditation for 17.7 min (SD = 9.19 min) each day. In addition, they engaged in ethical living exercises, on average, on 51.6 out of 60 days, corresponding to 86% of all days (M = 0.86, SD = 0.35). Hence, the participants generally showed high adherence to a home practice. Practicing physical yoga was rated as significantly easier (M = 3.48, SD = 1.06) than practicing mantra meditation (M = 2.92, SD = 1.14), with *t* (964) = 15.34 and *p* < 0.001, or ethical living (M = 2.99, SD = 0.99), with *t* (841) = 13.17, *p* < 0.001. Course adherence was high, with most participants attending seven to eight sessions and only one participant attending four sessions. The latter participant also responded irregularly to the daily questionnaire, did not answer the post questionnaire, and did not practice regularly.

### 3.3. Average Pain

**Figure 2:**
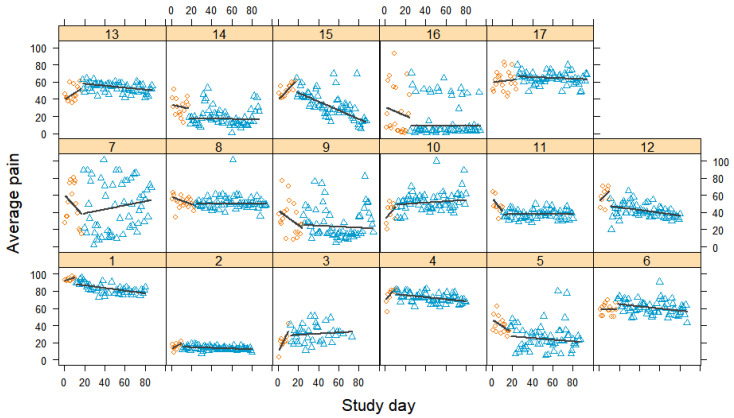
Average pain scores during baseline and treatment for each participant, with regression lines for each phase. Orange circles represent baseline measurements, blue triangles treatment measurements, black lines regression lines, and numerals labels indicate each case.

Figure 2 shows the individual changes in average pain levels over time. As pain should decrease over time, a downward trend would represent an effect in the expected direction. Measurements were taken daily so that the baseline phase comprised a maximum of 10, 17, or 24 measurement points. The treatment phase comprised a maximum of 60 measurement points.

Participants varied in the daily fluctuations in their average pain levels. While for some participants, their pain levels remained fairly consistent, for others, they fluctuated quite strongly, particularly for the patients suffering from chronic migraines (cases 3, 7, and 16). Three quarters of the participants exhibited either decreases or increases in average pain levels during the baseline phase. In the treatment phase, the average pain levels improved for more than half of the participants. The pain reductions mostly followed a linear trend, with recurring pain spikes for some participants (cases 5, 6, 9, 14, 15, and 16). For some participants, perceived pain first increased at the beginning of treatment and then decreased, followed by mostly returning to baseline levels (cases 6, 13, and 17). We observed consistent increases in pain levels for participants 3 and 10; however, for the former, the variations in pain levels appeared to decrease over time.

**Figure 3:**
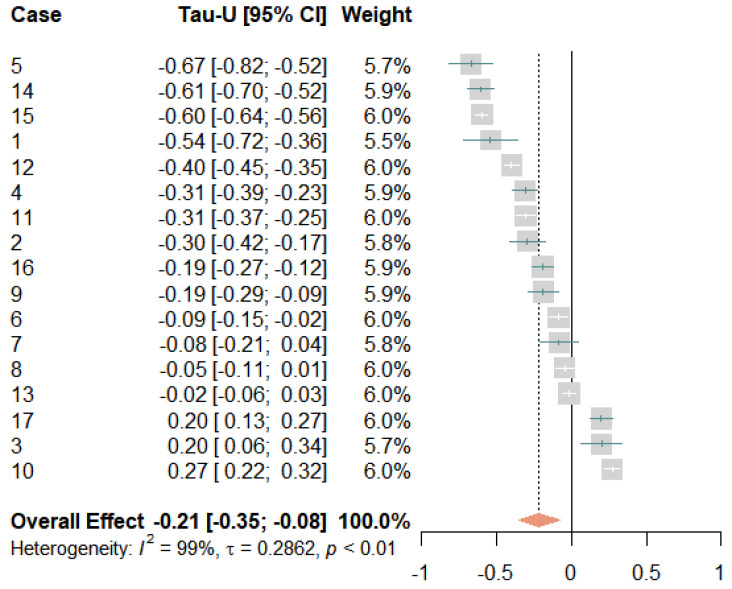
Forest plot of the average pain levels. The grey squares indicate the weight of each case with the mark in the middle representing the observed effect size and the corresponding line the confidence interval. The orange diamond shows the pooled effect size and its length symbolizes the confidence interval.

Figure 3 shows the results of the meta-analysis for this variable in a forest plot. The orange diamond shows the weighted total effect size with corresponding 95% confidence intervals and indicates the average treatment effect, which was Tau-*U* = 0.21 [0.35; 0.08]. Hence, average pain showed a small significant improvement over time. Eleven effects yielded significant decreases in pain, three effects indicated significant increases, and three effects were non-significant. This variability was also reflected in the high heterogeneity measures (*I*^2^ = 99% and τ = 0.29, where τ was the standard deviation of the average effect size). As the study conditions and measurements remained constant across the participants, this heterogeneity could be attributed exclusively to the interindividual differences between the participants. Hence, although the average treatment effect was significant, we had to assume that not all participants benefitted equally well from the treatment.

### 3.4. Strongest Pain

**Figure 4:**
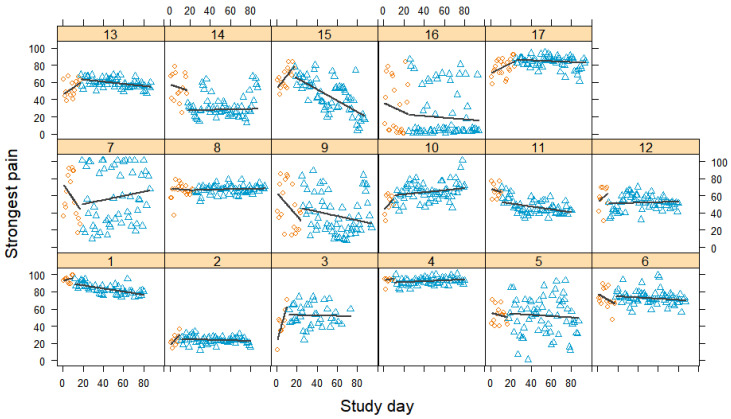
Strongest pain scores during baseline and treatment for each participant, with regression lines for each phase. Orange circles represent baseline measurements, blue triangles treatment measurements, black lines regression lines, and numerals labels indicate each case.

**Figure 5:**
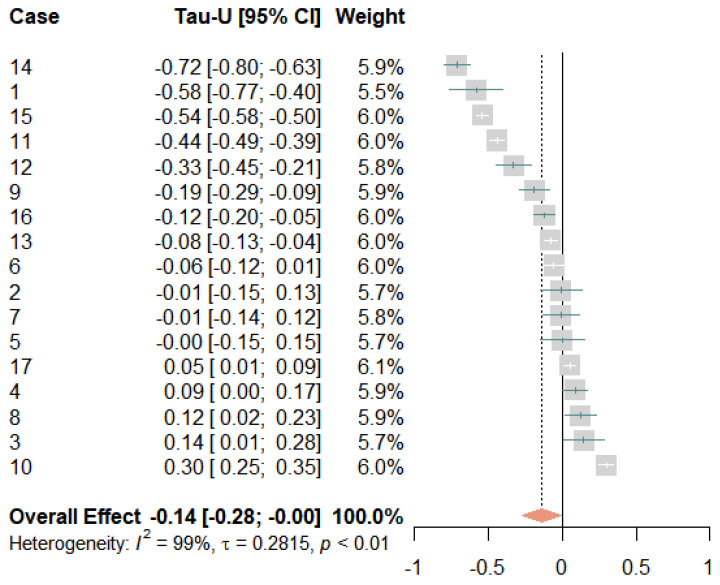
Forest plot of the strongest pain levels. The grey squares indicate the weight of each case with the mark in the middle representing the observed effect size and the corresponding line the confidence interval. The orange diamond shows the pooled effect size and its length symbolizes the confidence interval.

Figure 4 shows the individual changes in the strongest perceived pain levels over time. These corresponded to the patterns observed for average pain, albeit on a generally higher level. We saw similar increases and decreases in the baseline phase but with slightly smaller treatment effects. Figure 5 shows the corresponding forest plot. Overall, the treatment had a small effect, with Tau-*U* = −0.14 [−0.28; −0.00]. Eight participants exhibited significantly small to very large reductions in their strongest pain levels. Four participants reported no changes in their strongest pain levels and five participants reported small to moderate increases. The latter included the three participants whose average pain levels increased, plus one participant who experienced no change in average pain (case 8) and one participant whose average pain decreased (case 4). Again, heterogeneity was very high (*I*^2^ = 99% and τ = 0.28), suggesting an inconsistent effect of the treatment.

### 3.5. Pain Self-Efficacy

**Figure 6:**
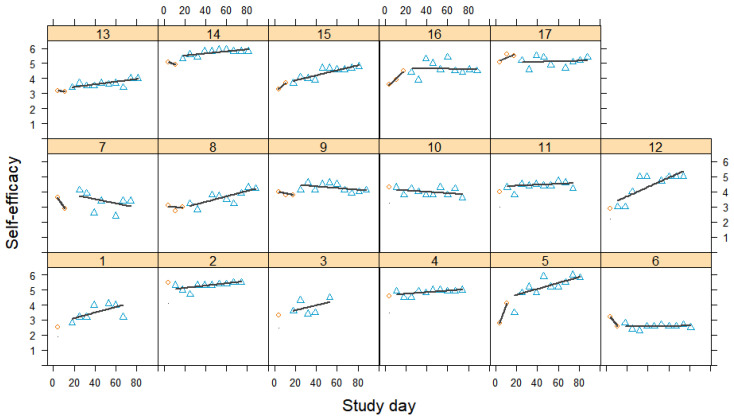
Self-efficacy scores during baseline and treatment for each participant, with regression lines for each phase. Orange circles represent baseline measurements, blue triangles treatment measurements, black lines regression lines, and numerals labels indicate each case.

Figure 6 depicts the development of participants’ pain self-efficacy ratings over the course of the study. Due to the weekly measurements, there were a maximum of three measurement points in the baseline phase and a maximum of ten in the treatment phase. Pain self-efficacy fluctuated, to some extent, in the baseline phase, but it was difficult to draw firm conclusions about trends as there were too few measurements. In the beginning of the intervention, most participants showed a gradual increase in pain self-efficacy ratings. For some, these increases were linear, while others fluctuated (to a larger extent). Overall, there were marked interindividual differences in the general levels and slopes of the curves. 

**Figure 7:**
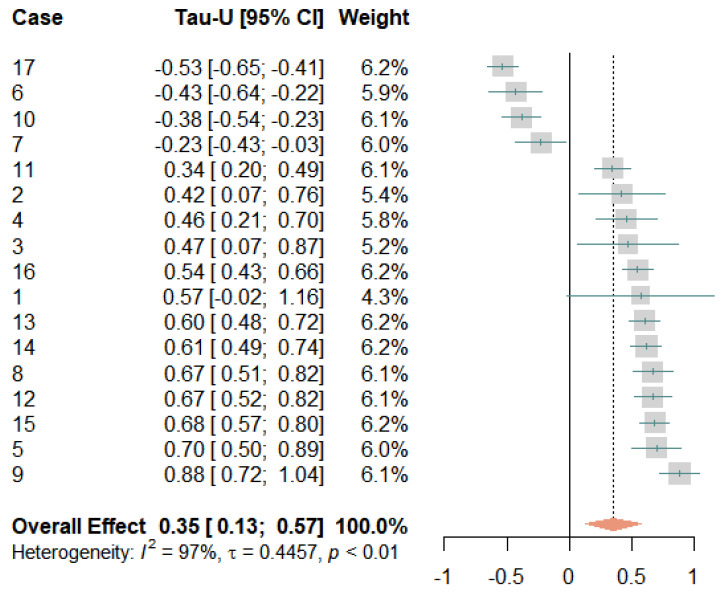
Forest plot of the self-efficacy ratings. The grey squares indicate the weight of each case with the mark in the middle representing the observed effect size and the corresponding line the confidence interval. The orange diamond shows the pooled effect size and its length symbolizes the confidence interval.

Figure 7 presents the results of the meta-analysis. With a mean Tau-*U* of 0.35 [0.13, 0.57], the overall effect showed a significant medium-sized improvement in the pain self-efficacy rating. Twelve effects yielded significant results, with moderate to very large effect sizes in the expected direction. Four effects were significantly negative, and one effect was non-significant. The negative effects were found for two participants who also experienced increased pain (cases 10 and 17), plus two participants whose pain levels did not change much (cases 6 and 7). A very large amount of variation was not accounted for (*I*^2^ = 97% and τ = 0.45), suggesting strong variations in the responses to the treatment.

### 3.6. Wellbeing

**Figure 8:**
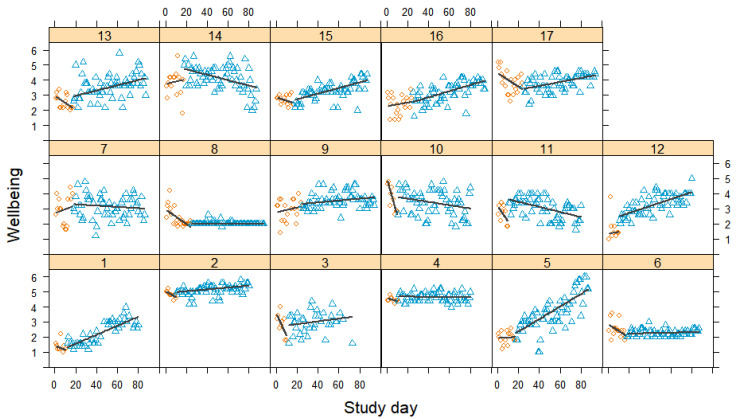
Wellbeing scores during baseline and treatment for each participant, with regression lines for each phase. Orange circles represent baseline measurements, blue triangles treatment measurements, black lines regression lines, and numerals labels indicate each case.

**Figure 9:**
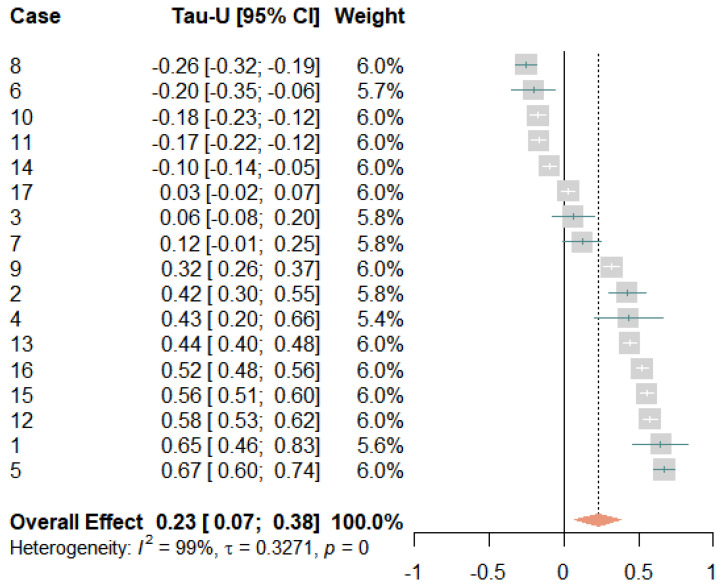
Forest plot of the wellbeing measurements. The grey squares indicate the weight of each case with the mark in the middle representing the observed effect size and the corresponding line the confidence interval. The orange diamond shows the pooled effect size and its length symbolizes the confidence interval.

Figure 8 displays how daily wellbeing developed over time for each participant. In both phases, wellbeing fluctuated day to day, with some participants reporting stronger daily variations than others. Most participants showed either a negative trend or no trend in the baseline phase. In contrast, more than two-thirds of the participants experienced a gradual and linear improvement in wellbeing during the treatment compared to the baseline. Only a few of the participants showed no or small negative effects. The meta-analysis yielded a significant mean effect, with Tau-*U* = 0.23 [0.07, 0.38], suggesting that wellbeing improved to a moderate extent (see Figure 9). Nine effects yielded significant results, with moderate to very large effect sizes in the expected direction. Three effects were non-significant and five effects indicated significant small decreases in wellbeing. Only two of these participants (cases 8 and 10) had also reported increased pain—one had experienced reduced self-efficacy (case 6) and the other two had reported positive effects (cases 11 and 14). Again, a large amount of variation was not accounted for (*I*^2^ = 99% and τ = 0.33).

### 3.7. Possible Explanations for the Findings

Chronic pain is often affected by life circumstances, and our participants readily reported daily events and reflections in the questionnaires. We have analyzed these qualitative statements to elaborate on and find possible explanations for our reported results. On average, the participants were very satisfied with and deeply grateful for their course and had continued to practice until follow-up. Although not all participants experienced reductions in pain, during follow-up, most stated how they had learned important lessons for their lives. These included perceiving and respecting their needs, behaving more kindly and mindfully to themselves and others, and finding ways to deal with or alleviate their pain.

Two of the three participants who suffered from migraines (cases 3 and 7) did not benefit from the treatment regarding pain or wellbeing, but they experienced increased levels of self-efficacy. They reinforced this finding in their qualitative statements at the end of the study. Participant 7 experienced a range of very stressful life events throughout the course of the study, including multiple deaths of family members and friends. Her pain scores fluctuated the most and she was the only participant who repeatedly marked the highest pain score (100). She stated that the course was very helpful for her during this difficult time.

Participants 10 and 17 exhibited deteriorations in almost all variables over time. Nevertheless, both of them qualitatively stated that they had benefitted from the course and the practices as these helped them to find calmness and change their perspective. Interestingly, while participant 10 reportedly noted work stress or family problems throughout the study, participant 17 reported a lot of positive events such as family visits or participating in musical events. Participants 6 and 8 experienced worsening in some but not all variables. While participant 8 reported some family- and work-related difficulties during the study, participant 6 experienced a throwback during follow-up due to the sudden death of a loved one. Both expressed gratitude for the course and experienced the practices as very helpful.

Moreover, other participants in our sample were exposed to a wide range of stressful life events that had negative impacts on their quality of life. These events included a depressive episode (case 11), the death of a loved one (cases 13 and 14), and the deterioration of an illness (case 14). Nonetheless, many participants perceived the treatment as beneficial and stress-relieving. During the last two weeks of the treatment, participant 9 entered a day clinic to treat her pain. Therefore, an amelioration of her symptoms could not be attributed solely to MBLM.

In contrast, the strongest pain decreases were reported by patients with chronic back pain. Particularly participants 1, 5, 14, and 15 benefitted from the treatment across variables. In the qualitative statements, they expressed great enthusiasm for the course and how its’ different elements helped and inspired them in numerous ways. They reported being calmer and more mindful and present, and some described profound changes in their perspectives after engaging with the ethical component of MBLM.

In addition, we explored the relation between the amount of home practice and the outcomes. The duration of home yoga practice was moderately related to reductions in the strongest pain levels (*r* = −0.42), and it showed small correlations with reduced average pain levels (*r* = −0.23) and improved self-efficacy levels (*r* = 0.24) but no correlation with quality of life (*r* = −0.08). Likewise, the duration of meditation practice was correlated with reduced strongest pain levels (*r* = −0.25) but not with average pain levels (*r* = −0.08). Surprisingly, meditation practice exhibited small negative correlations with self-efficacy levels (*r* = −0.21) and quality of life (*r* = −0.12). In contrast, the engagement in ethical living activities was related to small improvements in all variables (strongest pain (*r* = −0.13), average pain (*r* = −0.14), self-efficacy (*r* = 0.17), and quality of life (*r* = 0.24)). Hence, the more participants engaged in home yoga or ethical practice, but not necessarily meditation practice, the better they responded to the treatment.

## 4. Discussion

The current study provided further insight into the effects of MBLM as a second-generation mind-body intervention [50], complementing the results published by Matko et al. [35,51] and Bringmann et al. [34,52]. It evaluated MBLM’s effects on pain-related outcomes in outpatients suffering from chronic pain using a single-case multiple-baseline design. The intervention moderately enhanced the participants’ pain self-efficacy ratings and their average pain and wellbeing levels, and it slightly reduced their strongest pain levels. However, the participants’ responses were heterogeneous, indicating large interindividual differences. Interestingly, the treatment was perceived as very helpful by most participants, even if the experienced pain or wellbeing did not improve. In addition, the home yoga and ethical practices were related to improved outcomes, whereas the home meditation practice was only related to reduced strongest pain levels. The current results consolidated previous knowledge on the effectiveness of yoga interventions in improving pain self-efficacy, wellbeing, and pain perceptions [21,23,24]. Yet, as reactions to the intervention varied and we observed a relatively high dropout rate, we concluded that it did not help all participants equally well, and thus, it does not necessarily represent a good addition to interdisciplinary multimodal pain therapy.

MBLM teaches classical yoga, incorporating its ethical and philosophical roots and providing participants with a range of beneficial practices. As such, it differs from most previous studies, which mostly neglected the aspect of yogic philosophy [29,53]. Furthermore, because of the heterogeneity of yoga interventions and differences in study designs, it is difficult to compare our results with those of previous studies. Nevertheless, this study complements and extends previous research.

### 4.1. Beneficial Effects of MBLM

Previous studies substantiated how yoga interventions improved pain self-efficacy, to a significant extent [27,54,55]. Likewise, high pain self-efficacy positively influenced perceived pain intensity [54,56,57]. As yoga interventions do not always result in pain reductions for all chronic pain patients [16,26,27], improving patients’ pain self-efficacy can change their internal engagement and, as a result, their relationship to pain itself. The accompanying change in body awareness can help to manage the common fear of physical activity and the day-to-day management of chronic pain [27,54,56]. This corresponded with the qualitative statements of the participants in this study. Pearson et al. [16] hypothesized that incorporating the philosophical and spiritual components of yoga may induce better and more lasting effects on self-efficacy and physical and mental health. The current study provides evidence for this notion as the treatment included the ethical component of yoga and elicited large increases in self-efficacy for a majority of the participants.

The results of this study were consistent with previous research that found small to moderate effects of yoga treatments on wellbeing and quality of life in pain patients [23,26,55], particularly for complex yoga interventions [16,58,59]. Tekur et al. [26] proposed that practicing meditation could lead participants to practice mindfulness and avoid emotional overreactions to stress in the future. Although the dismantling trial on MBLM underpinned that mantra meditation may be the driving force in improving participants’ emotional regulation and body awareness skills [51], it substantiated that the ethical component was crucial for increasing wellbeing [35]. This is in line with the present study, which suggests that engaging in yoga or ethical exercises might be more beneficial and/or feasible in this respect than meditating.

### 4.2. Participants Differed in Their Response to the Treatment

The effects on perceived pain were small and inconsistent. This contrasted with previous studies that reported moderate to large effects on pain reduction from yoga interventions. However, many of these interventions focused on specific conditions, such as migraines, rheumatoid arthritis, or chronic low back or neck pain [24,60,61,62]. Reasons for the varying strength of the effects could depend on the type, location, or cause of the chronic pain, comorbidities, or yoga style [27,63,64]. In the present study, we found stronger effects for patients with back pain compared to patients with migraines. This corresponded with earlier studies that did not find a positive effect of yoga on migraine pain intensity [65,66]. However, in our study, there was one participant with migraines who did benefit from the treatment. This underpins the need to take into account the great variability between participants that we observed. People differ in their pathology, their individual preferences and needs, and their resilience to challenging life events [67,68,69]. Accordingly, the participants in our sample who faced similar stressful life events responded quite differently with respect to their perceived pain and wellbeing over time. Hence, clinical practice and research should consider these interindividual differences in the multimodal treatment of pain and aim to develop and evaluate personalized treatments [41]. This personalization should go beyond personalized medication and include complementary mind-body interventions.

### 4.3. Limitations

The study had several limitations. For self-efficacy, there were only few measurements in the baseline phase, which could lead to an overestimation of effect sizes. Despite this limitation, the TAU-*U* remains one of the most common and valid methods for effect size estimation [46]. Another limitation concerns the female-only participants, which limited the generalizability of the results. Our initial sample included two men who, unfortunately, dropped out of the study. In general, women are at higher risk for many common pain conditions and are more sensitive to pain, but they also appear to benefit more from multimodal pain treatment [44]. Nevertheless, future studies should try to recruit both men and women to evaluate this type of intervention. Furthermore, we had to assume that the participants were intrinsically motivated and interested in yoga and meditation. The drop-outs included two participants who, according to the therapists, were not responding well to the intervention or were not experiencing the quick relief they had hoped for. Hence, clinicians should consider individual patient characteristics, such as openness and patience, when prescribing MBM practices and should not make exaggerated claims regarding their efficacy.

Nevertheless, this study was the first study to evaluate the feasibility of the MBLM program in chronic pain, and further studies with larger sample sizes are warranted. In addition, the diagnosis and duration of pain varied among the participants from a few months to longer than five years. Many participants had long histories of pain treatments and stated their eagerness to try out a new intervention in the hope that it could help. Chronic pain is a complex syndrome that requires multimodal treatment, and sometimes pain management represents a more realistic goal than achieving freedom from pain. In this context, larger gains in pain self-efficacy than in pain reduction might be a promising pathway. Future studies could be limited to a specific type of chronic pain in order to make clearer statements about when and where MBLM could be a helpful adjunct treatment.

## 5. Conclusions

The present findings support the efficacy of yoga interventions in chronic pain and demonstrate the potential of the comprehensive approach of Meditation-Based Lifestyle Modification in the treatment of chronic pain. The ethical and philosophical aspects of classical yoga proved to be valuable treatment components and should be further explored in controlled studies to verify their therapeutic utility in multimodal pain management [70,71].

## Figures and Tables

**Table 1:** Case descriptions.

Case	Baseline	Age	Occupation	Medical Diagnoses	Duration of Pain
1	10	39	Unemployed	Fibromyalgia	>5 years
2	10	65	Employee	Lower back pain and knee arthrosis	1–2 years
3	10	19	Student	Migraines and depression	>5 years
4	10	27	Trainee	Chronic intractable pain, depression, and anxiety disorder	2–5 years
5	17	55	Employee	Lumbar and other intervertebral disc disorders with radiculopathy and depression	6–12 months
6	17	66	Retired	Chronic pain and headaches	>5 years
7	17	57	Employee	Migraines, vascular headaches, and atypical facial pain	>5 years
8	24	51	Employee	Spinal stenosis	>5 years
9	24	49	Employee	Chronic pain and burnout	>5 years
10	10	47	Employee	Lumbar and other intervertebral disc disorders with radiculopathy and anxiety disorder	2–5 years
11	10	68	Retired	Lumbar and other intervertebral disc disorders with radiculopathy and sleep disorder	>5 years
12	10	47	Employee	Cervical disc disorder and lumbar and other intervertebral disc disorders with radiculopathy	6–12 months
13	17	62	Retired	Chronic back pain, scoliosis, and burnout	>5 years
14	17	54	Self-employed	Interstitial cystitis, other chronic cystitis, lower back pain, and psychological factors associated with chronic pain	2–5 years
15	17	60	Employee	Lumbar and other intervertebral disc disorders with radiculopathy	6–12 months
16	24	34	Homemaker	Migraines	>5 years
17	24	79	Retired	Lower back pain	2–5 years

## Data Availability

The data that support the findings of this study are available on request from the corresponding author (H.C.B.). The data are not publicly available due to the sensitivity of human data, which could compromise the research participant privacy/consent.
